# Video‐rate tunable colour electronic paper with human resolution

**DOI:** 10.1038/s41586-025-09642-3

**Published:** 2025-10-22

**Authors:** Ade Satria Saloka Santosa, Yu-Wei Chang, Andreas B. Dahlin, Lars Österlund, Giovanni Volpe, Kunli Xiong

**Affiliations:** 1 Department of Materials Science and Engineering, The Angstrom Laboratory, Uppsala University, Uppsala, Sweden; 2 Department of Physics, University of Gothenburg, Gothenburg, Sweden; 3 Department of Chemistry and Chemical Engineering, Chalmers University of Technology, Gothenburg, Sweden; 4 Present Address: Department of Chemistry, Umeå University, Umeå, Sweden

**Keywords:** Metamaterials, Metamaterials

## Abstract

As demand for immersive experiences grows, displays with smaller sizes and higher resolutions are being viewed increasingly closer to the human eye^1^. As the size of emitting pixels shrinks, the intensity and uniformity of their emission are degraded while colour cross-talk and fabrication complexity increase, making ultra-high-resolution imaging challenging^2–4^. By contrast, electronic paper, which uses ambient light for visibility, can maintain high optical contrast regardless of pixel size, but cannot achieve high resolution^5,6^. Here we demonstrate electronic paper with electrically tunable metapixels down to ~560 nm in size (>25,000 pixels per inch) consisting of WO_3_ nanodisks, which undergo a reversible insulator-to-metal transition on electrochemical reduction. This transition enables dynamic modulation of the refractive index and optical absorption, allowing precise control over reflectance and contrast at the nanoscale. By using this effect, the metapixels can achieve pixel densities approaching the visual resolution limit when the display size matches the pupil diameter, which we refer to as retina electronic paper. Our technology also demonstrates full-colour video capability (>25 Hz), high reflectance (~80%), strong optical contrast (~50%), low energy consumption (~0.5–1.7 mW cm^–^^2^) and support for anaglyph 3D display, highlighting its potential as a next-generation solution for immersive virtual reality systems.

## Abstract (web summary)

Researchers have developed retina electronic paper with electrically tunable metapixels comprising WO_3_ nanodisks, achieving >25,000 pixels per inch, full-colour video and high contrast, enabling low-power immersive displays for virtual reality applications.

## Main

**Fig. 1:**
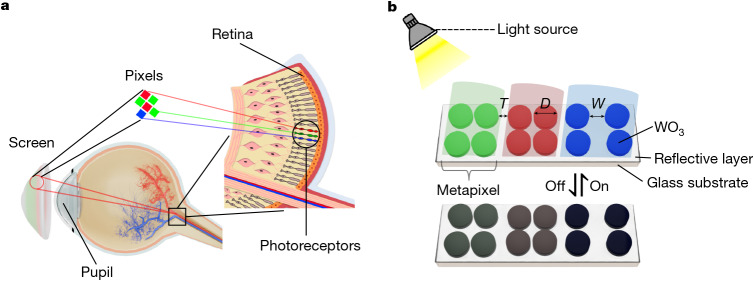
Schematic of retina E-paper. **a**, Conceptual illustration of an ultimate virtual reality display. The display is sized to approximate the human pupil and features an ultra-high pixel density serving as a conceptual benchmark inspired by the retina, supporting ultra-fine visual detail. **b**, Structural diagram of metapixels (subpixels). The metapixels consist of WO_3_ nanodisks and a reflective layer on a glass substrate. By varying *D* and *W* of the nanodisks, the metapixels can selectively reflect RGB colours. Further tuning of *T* enables the generation of hybrid colours such as CMY. As WO_3_ is electrochromic it can undergo reversible electrochemical reactions, yielding reflectance modulation of the WO_3_ nanodisks, enabling an RGB video display.

From cinema screens and televisions, to smartphones and virtual reality headsets, displays have progressively moved closer to the human eye, featuring smaller sizes and higher resolutions. As display technology advances, a fundamental question arises regarding the ultimate limits of display size and resolution. To provide an immersive and high-fidelity visual experience, the display is designed with a size comparable to the human pupil and an ultra-high pixel density. It establishes a conceptual benchmark inspired by the resolving limits of the retina (Fig. 1a) and defines a practical boundary for display technologies, which we term the retina display.

Assuming an effective display aperture of 8 mm, corresponding to the maximum human pupil diameter under scotopic conditions, and a field of view of 120°, consistent with the functional limits of human vision, achieving the maximum angular resolving capacity of approximately 60 pixels per degree would require a display pixel density of around 23,000 pixels per inch (PPI). This value represents a conceptual benchmark rather than a practical specification, because under typical photopic conditions with a smaller pupil size (~4 mm) the required pixel density would be even higher. In practice, however, as the display is not intended to be positioned directly in the pupil plane (because of safety considerations and to satisfy optical invariants such as étendue), the resolution requirement can be relaxed, because increasing the eye–screen distance effectively enlarges the screen size.

Unfortunately, as pixel sizes continue to shrink in mainstream emissive displays, diminished emitter dimensions lead to reduced brightness, compromised uniformity, increased colour cross-talk and greater fabrication complexity, posing considerable challenges for ultra-high-resolution imaging^1–3^. Currently, commercially available smartphone display pixels are typically around 60 × 60 μm^2^ (~450 PPI), which is approximately 2,500 times larger than the theoretical size required for the ultimate retina display. Already at this scale, the emitted light becomes difficult for the naked eye to perceive, particularly in bright outdoor environments. Moreover, the smallest published colourful micro-light-emitting-diode (micro-LED) display currently available achieves only a pixel size of 4 × 4 μm^2^ (excluding the distance between pixels)^4^, making it challenging to replicate retinal-level resolution across vast fields of view. Furthermore, at such small scales, colour cross-talk and uniformity remain considerable technical hurdles. These limitations expose the large challenges of using conventional emissive display technology to realize the ultimate virtual reality display.

Reflective displays, which rely on ambient light for visibility, do not have luminosity issues, and their optical contrast remains unaffected by pixel size reduction given that reflection is governed by the polarization of materials at the nanoscale. However, existing reflective display (electronic paper or E-paper) technologies are hampered by marked limitations. Reflective liquid crystal displays, for instance, are constrained by the thickness of the liquid crystal layer, whereas electrophoretic displays (such as the Kindle) are restricted by the size of their capsules^5,6^. So far no commercially available reflective display technology has achieved high resolutions of above 1,000 PPI.

Optical metasurfaces have demonstrated the capability to achieve ultra-high pixel densities of more than 10,000 PPI (~2.5 μm pixel size), with patterned nanomaterials capable of printing images at resolutions of up to around 100,000 dots per inch, approaching the optical diffraction limit^7–9^. However, most modern nanoprinting methods rely on static materials, such as metals or high-refractive-index dielectrics^10–13^. When applied to dynamic display systems (for example, meta-organic LED), these materials require modulation through micro-light sources, which are still affected by the inherent limitation of electromagnetic reduced intensity as resolution increases^3^. Furthermore, they are often affected by lateral light leakage between adjacent colour subpixels, limiting their ability to produce ultra-high-resolution images. In fact, static reflective displays are also influenced by interactions between neighbouring pixels, altering their optical properties at ultra-high pixel densities, making it challenging to use conventional red–green–blue (RGB) subpixel configurations for image display^13–15^.

In recent years, there has been growing interest in integrating dynamic and static materials to explore tunable nanophotonics systems. Particularly in the field of displays, hybrid nanomaterials—combining tunable conjugated polymers or semiconductors as colour modulators with metallic nanostructures—have demonstrated the ability to modulate the intensity or the reflected colours of subpixels^16,17^. These technologies significantly enhance the colour gamut, reflectivity and optical contrast of E-paper and enable video display functionality^18–21^. However, owing to limitations in structure, materials and fabrication methods, the pixel sizes of these hybrid nanomaterials remain in the range of tens to hundreds of micrometres, making it challenging to achieve ultra-high-resolution displays^22–24^.

Here we propose a conceptually new E-paper technology, termed retina E-paper, capable of achieving ultra-high resolutions exceeding 25,000 PPI (~560 nm), surpassing the theoretical human visual limit of 60 pixels per degree across a 120° field of view on an 8 mm screen. The retina E-paper comprises electrochromic WO_3_ metapixels, which undergo an insulator-to-metal transition during electrochemical insertion of, for example, alkali ions, allowing electrically dynamic control over optical properties such as refractive index and absorption. This transition enables tunable reflectance and contrast, which is critical for optimizing display performance. The basic colour generation principle builds on the hierarchical structuring of building blocks, Mie scattering and interference between the building blocks. This technology enables the practical construction of ultra-high-resolution displays (for example, exceeding 100 megapixels) within a compact area, paving the way for the ultimate virtual reality display.

The retina E-paper comprises electrochromic WO_3_ metapixels integrated with a highly reflective substrate (Pt/Al). Its normalized high reflectance (~80%) and optical contrast (~50%) remain unaffected by pixel size reduction, maintaining exceptional visibility even at pixel sizes as small as approximately 400 nm. To minimize interference between adjacent pixels, we carefully optimized the dimensions and spacing of the primary colour metapixels, enabling full-colour displays by precisely mixing RGB subpixels. Furthermore, the substrate (Al/Pt) exhibits excellent conductivity. By reducing the lateral distance between the working and counter electrodes to 500 nm and using short-pulse input signals, we achieved greater than 95% optical contrast modulation of the WO_3_ nano-pixels within 40 ms, supporting a video display of more than 25 Hz. This refresh rate is more than ten times faster than those of the previously reported fastest WO_3_-based electrochromic devices^25^. The short distance between the working and counter electrodes enhances the external electric field driving ion doping, and this nanodisk design significantly reduces the required amount and increases the reaction surface area of WO_3_ compared with the planar surface. Furthermore, unlike emissive displays that require constant power, retina E-paper features colour memory, consuming energy mainly during pixel switching. Its power usage is around 1.7 mW cm^–^^2^ for video and around 0.5 mW cm^–^^2^ for static images, significantly less than that of other types of E-reader^26^.

Finally, to demonstrate the potential of retina E-paper for virtual reality applications, we use cyan, magenta and yellow (CMY) colour metapixels to reconstruct an anaglyph 3D butterfly image. Furthermore, to showcase its full-colour display performance, we reproduced a high-resolution image inspired by the iconic painting *The Kiss* by Gustav Klimt and dynamically modulated the colours using electrical control. The retina E-paper features a compact surface area of around 1.9 × 1.4 mm^2^ (about 1/4,000th the size of a standard smartphone display) while achieving an impressive resolution of 4,300 × 700 pixels.

**Extended Data Fig. 1:**
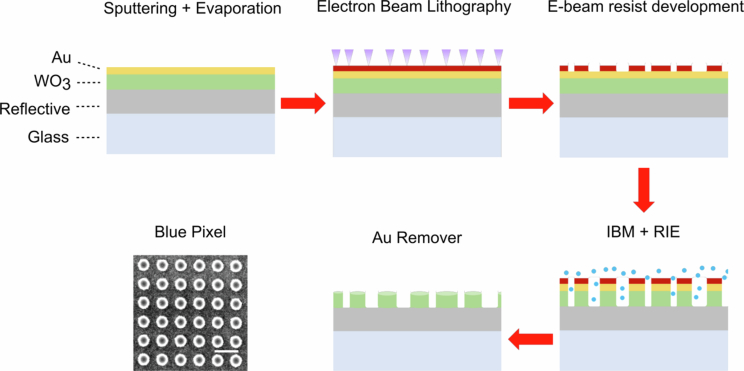
Nanofabrication of WO3 nanodisks. The reflective layer (Al/Pt), Au layer were deposited by e-beam evaporation and the WO_3_ layer was deposited by RF magnetron sputtering. After spin-coating an e-beam resist, nanopatterns are defined through e-beam lithography and developed to form structured resist templates. The patterns are then transferred onto the WO_3_ layer using ion beam milling (IBM) and reactive ion etching (RIE). Finally, the Au mask is removed by wet etching. An SEM image of a blue WO_3_ nanodisk pixel is presented as an example. Scale bar: 400 nm.

Figure 1b illustrates the fundamental structure of the retina E-paper, composed of electrochromic WO_3_ metamaterials integrated with a highly reflective (Al/Pt) substrate. In the bright state, WO_3_ behaves as a dielectric material with a high refractive index ranging from around 2 to 2.4 in the visible spectrum^27^, enabling the generation of high-resolution colours even at sizes smaller than the incident wavelength^11^. By precisely tuning the diameter (*D*) and spacing (*W*) of the nanodisks, the scattering modes can be adjusted to reflect the primary colours, RGB, forming the subpixels of one pixel of the retina E-paper. As the subpixels are made of meta-material, they are also called metapixels; however, optical interactions between these nanodisk-based subpixels can also affect colour mixing. Further tuning of the subpixel spacing (*T*) is required to ensure proper additive colour blending for display applications. After patterning the RGB subpixels, the next step is intensity modulation. As WO_3_ is an electrochromic material, meta-atom absorption can be dynamically modulated by electrochemical reactions under applied voltage, altering the reflectivity of the subpixels. This capability enables the retina E-paper to achieve dynamic colour modulation for display applications. The nanofabrication process is shown in Extended Data Fig. 1.

**Fig. 2:**
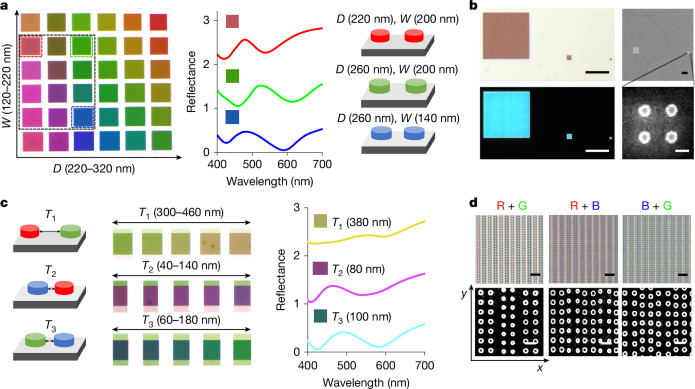
Design and characterization of WO_3_ metapixels. **a**, Metapixel design. Left: tuning *D* and *W* of WO_3_ nanodisks achieves a diverse colour palette. The dashed box highlights selected RGB pixels along with their intermediate regions, which contain CMY pixels. Middle: reflectance spectra of the selected RGB pixels. Right: corresponding *D* and *W* values for the chosen RGB pixels. **b**, Microscopic and structural characterization. Left: bright-field (top) and dark-field (bottom) microscope images of a red pixel with feature sizes of 20 μm, 2 μm and 420 nm, captured under ×100 magnification. Scale bars, 10 μm. Right: SEM images of 2 μm and 420 nm red pixels. Scale bars, 2 μm (top) and 200 nm (bottom). **c**, Colour mixing by subpixel arrangement. Left and middle: reflective colour varies as a function of *T* between adjacent RGB subpixels. Right: reflectance spectra of hybrid CMY pixels, corresponding to optimized subpixel spacing. **d**, High-resolution colour imaging. Top: bright-field microscope (×100) images of hybrid CMY pixels. Scale bars, 1 μm. Bottom: SEM images of the corresponding hybrid pixels. Scale bars, 500 nm. Source Data

Figure 2a demonstrates how the reflective colours of WO_3_ metapixels vary at a fixed thickness of 110 nm while varying the nanodisk diameter from 220 nm to 320 nm and the spacing from 100 nm to 200 nm. The thickness was chosen to balance Mie-resonance-based colour purity and electrochemical switching speed. Thinner layers may accelerate switching but reduce optical confinement and weaken Mie scattering, thus degrading colour saturation. This geometry range enables the metapixels to cover the entire visible spectrum. However, it is essential to note that not all RGB pixels are suitable for subpixels in retina E-paper. The reflected colour of each subpixel in an ultra-high-resolution E-paper system is influenced not only by its geometry but also by interactions with adjacent pixels (Fig. 2c). Therefore, selecting appropriate RGB pixels and ensuring that their hybrid reflected colours adhere to the principles of additive colour mixing is a critical step for achieving full-colour displays. In Fig. 2a (right), the spectra and corresponding geometries of the selected RGB pixels are presented: R (*D* = 220 nm, *W* = 200 nm), G (*D* = 260 nm, *W* = 200 nm) and B (*D* = 260 nm, *W* = 140 nm). The spectra are normalized to the reflective layer to highlight the WO_3_ nanodisk colour-tuning by structural changes. Unlike emissive displays, in which visibility diminishes with pixel size reduction, E-paper technology maintains consistent brightness and reflectivity even at ultra-high resolutions. As illustrated in Fig. 2b, the red pixel retains its colour and reflectance in both bright- and dark-field microscope images, even as the size is reduced from 20 μm to 420 nm. A minimum of four nanodisks per pixel is required to preserve the Mie scattering and grating modes of the nanodisks, resulting in minimum pixel sizes of 420 nm for red, 460 nm for green and 400 nm for blue.

**Extended Data Fig. 2:**
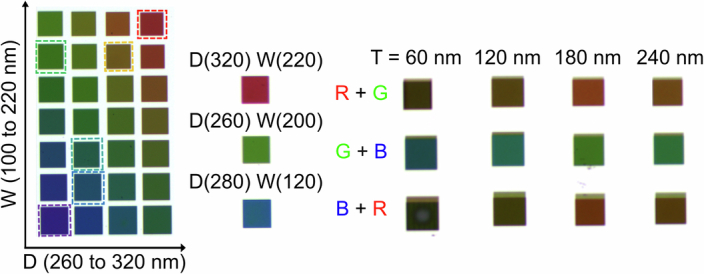
Arbitrarily selected combinations of RGB pixels. The intermediate regions of RGB pixels contain only cyan and yellow, with no magenta. By optimizing subpixel spacing (T), a yellow pixel appears when the red-green subpixel distance is 240 nm, while a cyan pixel emerges at a blue-green subpixel distance of 120 nm. However, regardless of the red-blue subpixel distance, magenta cannot be produced, highlighting a fundamental limitation in colour.

Once the smallest dimensions for the three primary colour metapixels are determined, the next step is merging them to achieve a full-colour display. In Fig. 2c, the grating modes between adjacent subpixels are influenced by the spacing (*T*) between subpixels, which changes reflected hybrid colours. According to the principles of additive colour mixing, the overlap between RGB should produce CMY, respectively. As shown in Fig. 2a, the intermediate region (black dashed box) between RGB pixels also contains CMY pixels. As long as the spacing between RGB subpixels is carefully designed, the grating modes of adjacent pixels can produce CMY colours, ensuring compliance with the additive colour principle. For comparison, Extended Data Fig. 2 presents several arbitrarily selected combinations of RGB pixels that fail to reproduce the CMY colours. After carefully selecting RGB pixels and tuning the inter-pixel spacing— 380 nm for red-green, 80 nm for blue–red and 100 nm for green–blue—the desired hybrid colours were successfully generated. The corresponding reflection spectra demonstrate that the reflectance of the hybrid-colour pixels matches that of the single-colour pixels.

Finally, Fig. 2d presents microscope and scanning electron microscope (SEM) images of the merged pixels producing CMY colours. Under high magnification (×100), the arrangement of alternating subpixels along the *x* axis to form hybrid colours is clearly visible. Notably, the Mie scattering mode of individual nanodisks is mainly determined by their size, whereas the grating mode of the subpixel arrays in the *x* direction governs the generation of reflective mixed colours.

**Extended Data Fig. 3:**
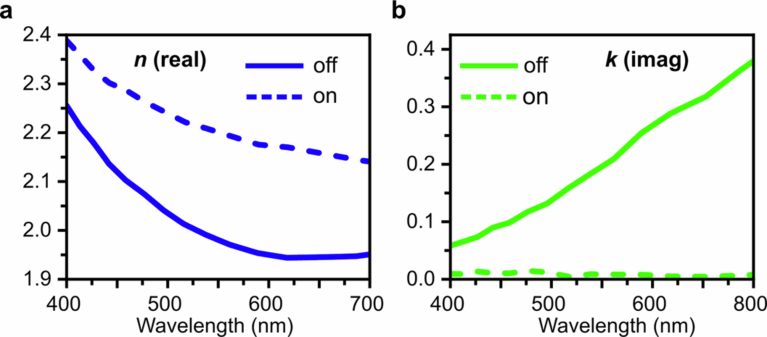
Refractive index of WO_3_ in the on and off states. **a**, Real (*n*) part of the refractive index of WO_3_ in the on and off states. **b**, Imaginary (*k*) part of the refractive index of WO_3_ in the on and off states. Source Data

As an electrochromic material, WO_3_ exhibits an electrically tunable refractive index (*n*) and extinction coefficient (*k*) across the visible spectrum (400–700 nm). In the insulator (colour) state, the refractive index varies from around 2.38 to 2.14, with *k*-values of <0.01. In the metal (black) state, *n* decreases from around 2.25 to 1.95, whereas *k* increases significantly (*k* > 0.4; Extended Data Fig. 3). This transition occurs on electrochemical reduction of WO_3_, when, for example, alkali ions or a proton, M^+^, are introduced into the material, altering its electronic structure. The reaction can be expressed as:

$${{\rm{WO}}}_{3}+x{{\rm{M}}}^{+}+x{{\rm{e}}}^{-}\to {{\rm{M}}}_{x}{{\rm{WO}}}_{3}$$

where M^+^ represents the doping ion (for example, Li^+^), *x* is the number of ions inserted into the WO_3_ structure (0 ≤ *x* ≤ 1) and e^−^ represents an electron. When WO_3_ is reduced, electrons are introduced into the conduction band comprising the W 5*d* bands. The injected electrons lead to the formation of localized polarons, which result in a transition from insulating (semiconducting) behaviour to increased conductivity or metallic-like behaviour as *x* increases and the polarons overlap. As a result, the refractive index decreases and the extinction coefficient increases, contributing to the optical contrast between the insulating (clear) and metallic (dark) states. Furthermore, the nanodisk structure of the WO_3_ metapixels, with its high refractive index (*n*), enables the concentration of incident light in the nanodisk. This effect further amplifies the light–matter interaction and absorption efficiency for stronger optical contrast.

**Fig. 3:**
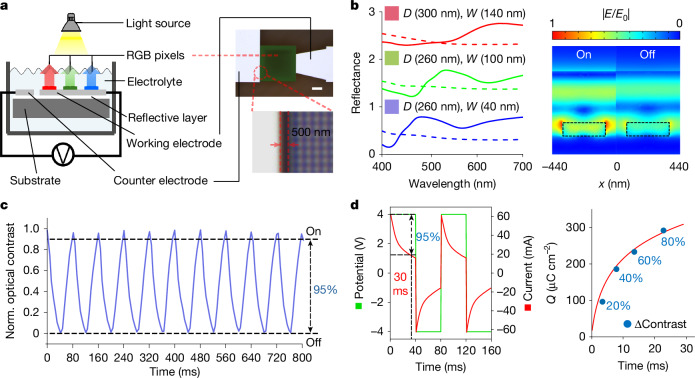
Electrochemical modulation of WO_3_ metapixels. **a**, Experimental set-up for electrochemical characterization. Left: cross-sectional schematic diagram illustrating the set-up used to characterize the optical properties of WO_3_ metapixels under electrochemical control. Right: microscope images of the characterized sample show a 500 nm gap between the working and counter electrodes, which enhances the local electric field for improved switching performance. Scale bar, 100 μm. **b**, Optical response of electrochemically controlled RGB metapixels. Left: reflectance spectra of the selected RGB metapixels in their electrochemically modulated on (sold lines) and off (dashed lines) states, demonstrating dynamic tunability. Right: numerical simulation of the reflectance modulation of red metapixels at a 650 nm incident wavelength, validating that most of the electric field is concentrated in the WO_3_ nanodisks. **c**, Switching speed characterization. By applying a short pulse voltage signal, 95% normalized optical contrast is achieved within 40 ms, demonstrating video-rate display applications. **d**, Electrical properties corresponding to optical modulation. Left: applied voltage pulses of ±4 V with a 40 ms duration produce corresponding current responses, with 95% of the current change occurring within 30 ms, indicating fast ion transport in the WO_3_ nanodisk structures. Right: relationship (turning off) between optical contrast change, response time and charge consumption. Owing to the colour memory effect of retina E-paper, video playback typically requires only minor updates, with intensity variations typically around 20% (equivalent to about 30 greyscale levels), thereby reducing the energy and time needed for dynamic content display. Source Data

Figure 3a presents the experimental set-up to electrochemically control the colour states of WO_3_ metapixels. The electrolyte consists of 1 M LiClO_4_ in acetonitrile, and the RGB pixel size is 350 μm. Metallic electrodes (Pt/Al) were used to minimize potential drops. Notably, we designed a lateral electrode configuration with a narrow 500 nm gap between the working and counter electrodes, enhancing the local electric field and significantly improving the switching speed^28,29^.

Figure 3b illustrates the measured normalized reflectance modulation of RGB metapixels in the on and off states. As both Mie scattering and grating modes are influenced by variations in the refractive index of the surrounding environment, the optimized nanodisk dimensions for the RGB metapixels are: R (*D* = 300 nm, *W* = 140 nm), G (*D* = 260 nm, *W* = 100 nm) and B (*D* = 260 nm, *W* = 40 nm). A clear distinction is observed when comparing the metapixel reflectance in air with that in electrolytes. Specifically, the reflectance in electrolytes is notably higher, whereas scattering in the red-light region is significantly suppressed. This effect arises because Mie scattering depends on the relative refractive index $m=\frac{{n}_{{\rm{particle}}}}{{n}_{{\rm{env}}}}$, where *n*_particle_ and *n*_env_ are the refractive index of the WO_3_ nanodisks and the surrounding environment, respectively. When *n*_particle_ is close to *n*_env_, the scattering is markedly reduced, making the material seem more transparent and desaturated in colour. Therefore, the colour contrast of WO_3_ metapixels in electrolytes is less saturated than in air; however, despite the ultra-thin WO_3_ nanodisks (~100 nm), the optical contrast remains at ~50%, significantly outperforming most planar WO_3_ electrochromic devices with the same thickness^30^. This enhancement is due to the high refractive index of WO_3_, concentrating the electric field of incident light in the nanodisks. After switching to the dark state, most of the incident light is absorbed, as confirmed by numerical simulations of red subpixel on and off states (Fig. 3b, right). The on–off switching of RGB pixels enables high-contrast modulation, which is essential for reflective display applications with full colour coverage.

**Extended Data Fig. 4:**
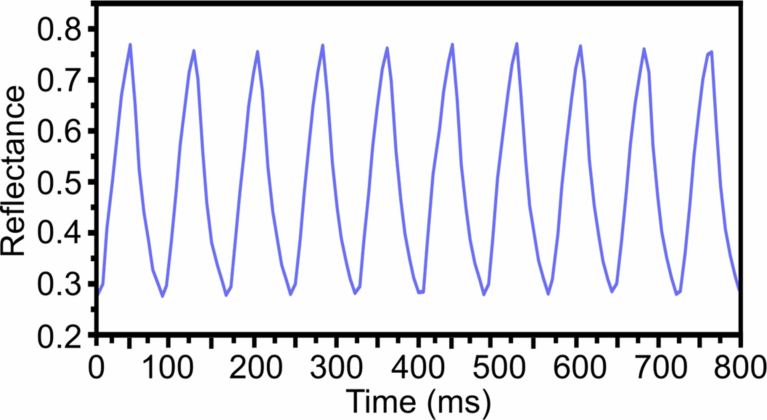
Optical contrast of red pixel. By applying a short pulse voltage signal, the reflectance change >0.48 is achieved within 40 ms. The maximum reflectance change under long-time constant voltage is ~0.5. Source Data

Benefiting from the strong local electric field between the closely spaced working and counter electrodes, as well as the ultra-thin amorphous WO_3_ nanodisks, yielding fast ion insertion^31^, the electrochemically tunable metapixels achieve an exceptionally fast switching time of only 40 ms to reach more than 95% of the total optical contrast modulation. Supplementary Video 1 demonstrates the rapid on–off switching of the red metapixel, confirming that metapixels support video display. Figure 3c shows ten cycles of reflectance variation in ±4 V pulse input signals. The normalized optical contrast is calculated by $\frac{R-{R}_{\min }}{{R}_{\max }-{R}_{\min }}$, where *R*, *R*_min_ and *R*_max_ are the real-time, minimum and maximum reflectance of the WO_3_ metapixels, respectively; this normalization clearly illustrates the change in optical contrast. The measured optical contrast is also shown in Extended Data Fig. 4. Notably, the operating voltage is comparable to the solid-state two-electrode WO_3_ electrochromic systems^32^. However, due to the significantly enhanced switching speed (>65-times faster), precise pulsed voltage control—rather than a constant bias—can be used to minimize energy consumption and mitigate side effects.

**Extended Data Fig. 5:**
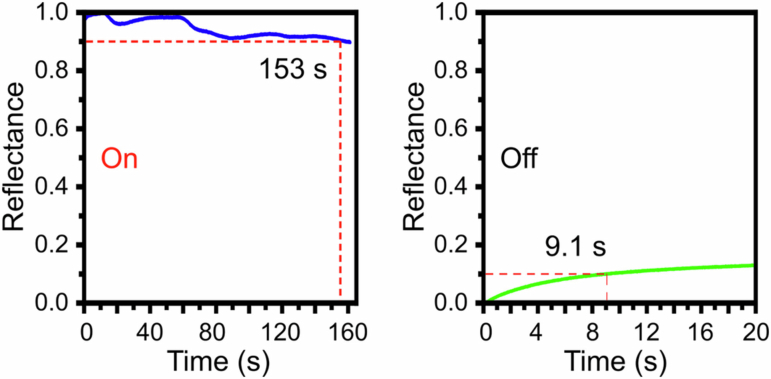
Colour memory effect. When the WO_3_ pixels are turned on, they can retain over 90% of reflectance for ~150 s without any additional power input. Conversely, after being turned off, the pixels maintain <10% reflectance for ~9 s, also without an external energy supply. Source Data

Retina E-paper offers ultra-low energy consumption due to its inherent colour memory effect. Energy is primarily consumed when the pixel intensity is actively changed. As shown in Extended Data Fig. 5, pixels in the on state can retain more than 90% of their reflectance for more than 150 s without any power input. This indicates that for relatively static pixels, a brief, low-energy signal applied every several tens of seconds is sufficient to maintain the displayed content. Figure 3d presents the electrical characteristics associated with colour modulation in retina E-paper. Figure 3d (left) shows the current response to a ±4 V voltage pulse, indicating that alkali ions can rapidly intercalate into the WO_3_ nanodisk layer at video rates, thereby modulating pixel colour states. Figure 3d (right) illustrates the relationship between optical contrast change, response time and charge consumption. Importantly, during video playback, only about 10% of pixels typically undergo state changes^20^, and among those, the average greyscale shift is around 30 levels^33^. This means that, on average, only approximately 20% normalized optical contrast is required for video display. Under such conditions, retina E-paper achieves an average switching time of around 5 ms (200 fps) and an energy consumption of about 1.7 mW cm^–^^2^. For static images, the energy use drops further to around 0.5 mW cm^–^^2^. This energy consumption is significantly lower than that of conventional electrophoretic displays and electrowetting displays^26^.

To further validate the display performance of retina E-paper, we fabricated metapixels to reproduce an anaglyph 3D butterfly and a high-resolution image inspired by *The Kiss*. The anaglyph butterfly demonstrates the feasibility of stereoscopic image rendering for virtual reality applications, whereas the reproduction of *The Kiss*—featuring intricate geometries and a wide colour gamut—highlights the suitability of retina E-paper for ultra-high-resolution, full-colour image display. As our substrate is a highly reflective material analogous to a white canvas, we used the CMY subpixels and used subtractive colour mixing to render the image. Importantly, the patterned image only demonstrates the display capability of WO_3_ metapixels. For the display application, a thin-film transistor (TFT) array should be used to independently control the reflectance of each pixel, whereas the background should be set to black. The image rendering should follow the additive colour principle using RGB subpixels (Fig. 2).

**Fig. 4:**
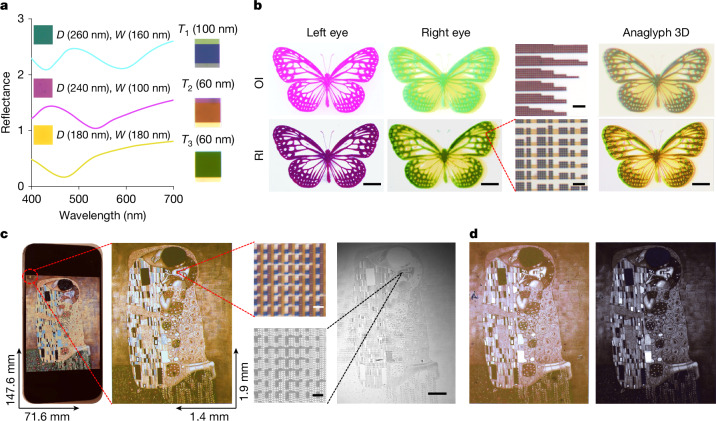
Characterization of retina E-paper display performance. **a**, Optical properties of CMY metapixels. Left: the reflectance spectra of the selected CMY metapixels demonstrate the spectral response. Right: photographs of the RGB pixels with optimized adjacent subpixel spacing for improved hybrid colour and display fidelity. **b**, Anaglyph 3D demonstration on retina E-paper. Left: the anaglyph stereo butterfly (Anaglyph 3D) original image (OI) decomposed into magenta (M) and cyan–yellow (CY) channels (top), and the corresponding reconstructed retina E-paper images (RI) for each eye (bottom). Scale bars, 200 μm. Middle: microscope images of individual M and CY channel pixels, illustrating submicrometre pattern fidelity. Scale bars, 2 μm. Right: full-colour anaglyph butterfly image: anaglyph 3D butterfly original image (top) and simulated retina E-paper reconstruction (bottom) demonstrating high-resolution 3D depth rendering. Scale bars, 200 μm. Original butterfly image licensed from Adobe. **c**, High-resolution display of *The Kiss* on retina E-paper versus iPhone 15. Photographs comparing the display of *The Kiss* on an iPhone 15 and retina E-paper. The surface area of the retina E-paper is ~1/4,000 times smaller than the iPhone 15. SEM and microscope images confirm that the displayed colours are generated by precisely arranged CMY subpixels. Scale bars, 2 μm (top and bottom left) 200 μm (right). Image of *The Kiss* reproduced with permission from Kingston Frameworks. **d**, Electrochemical display of *The Kiss* by retina E-paper. Photographs showing the display of *The Kiss* on retina E-paper in the on (left) and off (right) states, demonstrating reversible colour modulation when electrochemically tuned. Source Data

**Extended Data Fig. 6:**
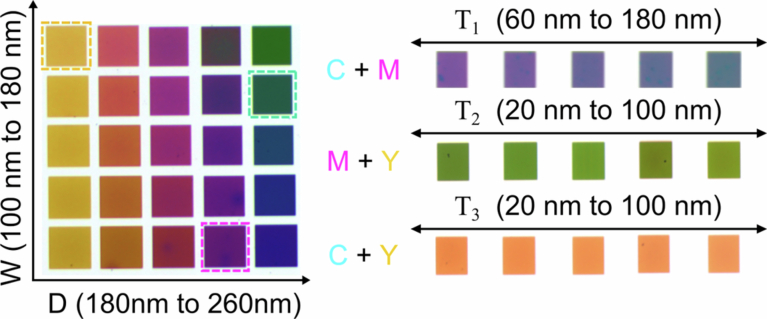
CMY pixels in the air. The intermediate regions of CMY pixels contain all RGB pixels. By optimizing subpixel spacing (T), a blue pixel emerges when the CM subpixel distance is 100 nm, green at an MY subpixel distance of 60 nm, and red at a CY subpixel distance of 60 nm.

Figure 4a (left) illustrates the nanodisk diameters and periodicities for the CMY metapixels alongside their corresponding reflection spectra, which show similar reflectance as RGB pixels. Figure 4a (right) presents the merged hybrid colour metapixels, in which the spacing between subpixels are B (*T*_1_ = 100 nm), R (*T*_2_ = 60 nm) and G (*T*_3_ = 60 nm). Notably, except for similar reflectance to RGB metapixels, the CMY system also ensures that the intermediate regions contain RGB pixels (Extended Data Fig. 6). The optimized nanodisk dimensions for CMY pixels are C (*D* = 260 nm, *W* = 160 nm), M (*D* = 240 nm, *W* = 100 nm) and Y (*D* = 180 nm, *W* = 180 nm).

**Extended Data Fig. 7:**
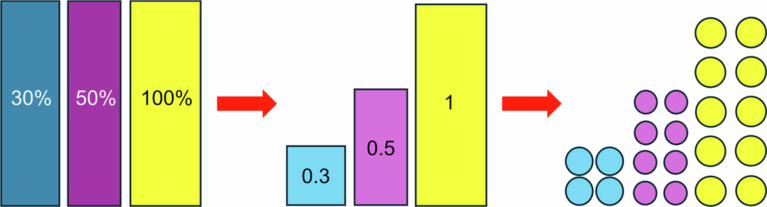
Transfer colour image to nanodisc pixels. The intensity of each CMY channel is converted into the height of its corresponding CMY rectangle as a fraction of the maximum value, indicating the proportion of light each CMY subpixel should reflect. The designated nanodisc arrays responsible for generating CMY colours are then arranged within these rectangles, approximating the intended subpixel colour composition.

Figure 4b illustrates an ultra-high-resolution anaglyph 3D display, achieved by encoding stereo image pairs (anaglyph 3D original image) into complementary colour channels—M for the left eye and CY for the right eye—and reconstructing them using the retina E-paper. This demonstration serves as a proof of concept for binocular disparity rendering, a fundamental mechanism underlying stereoscopic vision in virtual reality systems. As the retina E-paper does not connect to TFT arrays to individually adjust each subpixel, it achieves colour rendering solely through the precise geometric design of M–CY metapixels (Extended Data Fig. 7). By precisely reconstructing left and right eye images with submicrometre-resolution metapixels, the device successfully generates a full-colour 3D image (anaglyph 3D; Fig. 4b) through passive, compact optical configurations. This demonstrates an anaglyph 3D display with resolution exceeding 35,000 PPI (M) and 30,000 PPI (CY). Furthermore, this demonstration highlights the versatility of the retina E-paper platform, which can operate not only in full-colour mode but also in monochrome or dual-primary channel formats, broadening its applicability across a range of advanced display technologies.

Figure 4c presents a reconstructed full-colour image of *The Kiss*, directly comparing the retina E-paper with a commercial mobile-phone display (iPhone 15) in terms of both physical dimensions and display resolution. Whereas the iPhone screen measures 147.6 mm × 71.6 mm, the retina E-paper is only 1.9 mm × 1.4 mm, amounting to merely ~1/4,000th the area of the smartphone display. Despite this minuscule size, the retina E-paper achieves a resolution of 4,300 × 700, similar to the resolution of the smartphone display (2,556 × 1,179). Due to the inherent challenges of accurately controlling the reflectance of subpixels, as well as the narrower colour gamut compared with emissive displays, the perceived colour saturation of the retina E-paper is lower than that of the iPhone 15; however, this is the first demonstration of full-colour imaging achieved by three primary colour metapixels at such an ultra-high resolution. With an average pixel size of only around 560 nm, the display reaches an unprecedented >25,000 PPI, surpassing the resolution requirements for ultimate virtual reality displays. High-magnification microscope (×100) and SEM images (Fig. 4b, right) further confirm the well-ordered CMY metapixel arrangement and the vibrant colour rendering, validating the ultra-high resolution of the retina E-paper.

**Extended Data Fig. 8:**
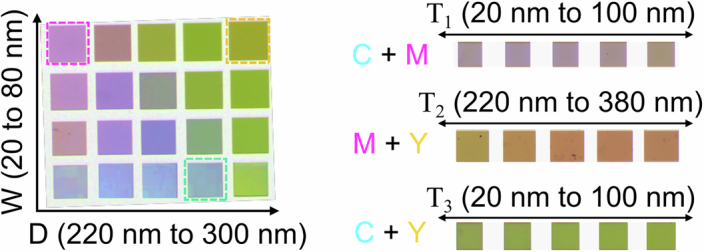
CMY pixels in the electrolyte. The intermediate regions of CMY colours contain corresponding RGB components. The subpixel distances are T_1_ = 40 nm for blue, T_2_ = 300 nm for red, and T_3_ = 60 nm for green.

**Extended Data Fig. 9:**
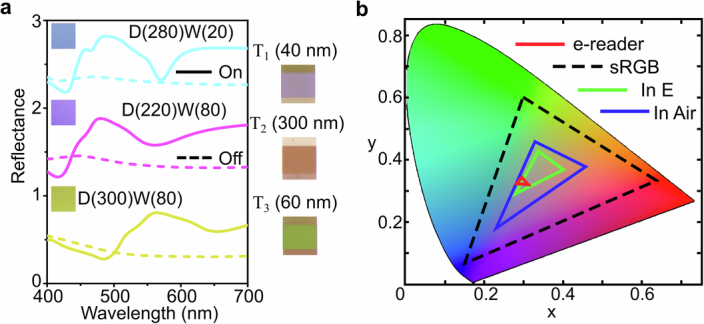
CMY colour modulation and comparison of colour performance with other devices. **a**, Electrochemical modulation of CMY meta pixels: Left: The Reflectance spectra of CMY metapixels in the electrochemically modulated on/off states demonstrate dynamic tuneability. Right: Photographs of RGB hybrid colours in an electrolyte environment with optimized adjacent CMY subpixel spacing. **b**, Comparison of colour gamut coverage: CIE chromaticity diagram comparing the colour coverage of an emissive display (sRGB), a commercial electrophoretic display (e-reader), and the Retina E-paper in both air and electrolyte environments. The results highlight the colour performance of the Retina E-paper relative to conventional display technologies. Source Data

To evaluate its electrically tunable colour performance, we reproduced *The Kiss* using CMY metapixels in an electrolyte environment. To maintain the presence of RGB pixels in the intermediate regions, the dimensions of the CMY metapixels were further optimized: C (*D* = 280 nm, *W* = 20 nm), M (*D* = 220 nm, *W* = 80 nm) and Y (*D* = 300 nm, *W* = 80 nm). The merged RGB subpixel spacing was adjusted accordingly: B (*T*_1_ = 40 nm), R (*T*_2_ = 300 nm) and G (*T*_3_ = 60 nm) (Extended Data Fig. 8). Extended Data Fig. 9a presents the corresponding reflection spectra of CMY pixels in their colour and dark states. Figure 4d showcases the photos of *The Kiss* under colour and dark states in the electrolyte. Owing to the weaker Mie scattering of WO_3_ nanodisks in an electrolyte environment compared with air, the displayed colours seem less saturated, with a noticeable reduction in extinction in the red region, resulting in an overall red-shifted colour. However, the system exhibits a distinct reflectance modulation between on and off states, highlighting its potential for dynamic display applications. Extended Data Fig. 9b compares the colour gamut coverage of commercial emissive displays, the retina E-paper in both air and electrolyte and the commercial colour electrophoretic display. Although the colour gamut of retina E-paper remains narrower than emissive displays, its performance in both air and electrolyte significantly surpasses a commercial colour E-reader^34^.

Currently, more than 80% of the information people perceive is through visual signals^35^. With the development of Internet-of-Things-based technology and increasing information transfer speeds, the demand for next-generation visual display technologies keeps growing. Retina E-paper not only reaches the theoretical resolution limit of human vision but also offers exceptional visibility. It enables full-colour video display while maintaining high reflectivity and optical contrast, which is promising for realizing ultimate virtual reality displays.

**Extended Data Fig. 10:**
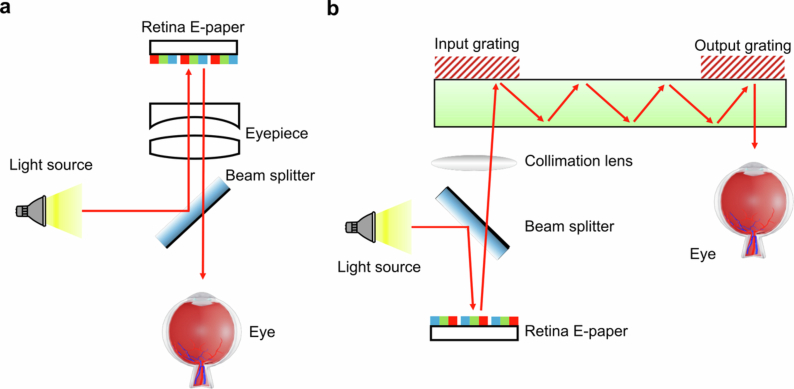
Front-illumination integration of Retina E paper into immersive AR/VR optics. **a**, Conventional VR headset configuration: Side-illumination is directed by a beam splitter through an eyepiece, focusing and magnifying the image onto the Retina E-paper. The reflected light retraces the path through the eyepiece and beam splitter into the eye. **b**, Meta-lens AR configuration: Side-coupled illumination is reflected by a beam splitter onto the Retina E-paper, then collimated and directed through a meta-lens optics module. After coupling into an input grating, the light undergoes internal waveguide propagation and exits through an output grating into the eye.

Unlike conventional emissive displays, retina E-paper devices require front-illumination to enable image visibility. Extended Data Fig. 10 illustrates two distinct optical architectures that accommodate this requirement: one compatible with conventional virtual reality headsets, and the other tailored to state-of-the-art, waveguide-based augmented reality–virtual reality lenses^36^. In fact, retina E-paper also holds significant potential for augmented reality applications, as it can leverage ambient light as the illumination source. This inherent compatibility with the environment enables natural visual integration, reducing reliance on light engines. Furthermore, as the primary illumination is provided by ambient light, its low power consumption enables substantial downsizing of the battery and even opens up the possibility of fully self-powered displays when combined with solar cells (with a typical output of ~15 mW cm^–^^2^).

Despite its advantages, retina E-paper requires further optimization in colour gamut, refresh rate, operational stability and lifetime. Lowering the operating voltage and exploring alternative electrolytes represent promising engineering routes to extend device durability and reduce energy consumption. Moreover, its ultra-high resolution also necessitates the development of ultra-high-resolution TFT arrays for independent pixel control, which will enable fully addressable, large-area displays and is therefore a critical direction for future research and technological development. Looking ahead, we anticipate significant advancements in this field and firmly believe that the evolution of the retina E-paper will ultimately influence everyone.

## Methods

### Nanofabrication of WO_3_ nanodisks

We fabricated WO_3_ nanodisk metamaterials (Extended Data Fig. 1) in a clean room using standard semiconductor processes. A 100 nm reflective layer (Al/Pt) and a 30 nm Au layer were deposited via electron beam evaporation (Lesker PVD 225). The middle 100 nm WO_3_ layer was deposited using radio frequency magnetron sputtering. After spin-coating the electron beam resist (MaN 2401, 3,000 r.p.m.), nanostructures were patterned by electron beam lithography (400 μC cm^–^^2^). The resist was developed in MF-24A for 45 s, followed by ion beam milling using Ar gas (80 mA, 200 V) for 2 min to etch the Au layer. Reactive ion etching with CF_4_ gas (100 W) for 5 min was then used to etch the WO_3_ layer. Finally, the Au layer was removed by wet etching. Samples were characterized using a Zeiss Supra 55 SEM for high-resolution imaging.

### Electrochemical measurements

Acetonitrile was purchased from Sigma-Aldrich, and LiClO_4_ was purchased from Fisher Scientific. The electrolyte contains 1 M LiClO_4_ in acetonitrile. A custom-built liquid cell with two electrode connections was used for electrochemical measurements. A potentiostat (Gamry Interface 1000) controlled the applied current and voltage. For the fast-switching measurements, a short 40 ms voltage pulse ranging from −4 V to 4 V was applied. Low constant voltages from −1 V to 1 V were applied for 2 min to obtain the reference optical contrast of WO_3_.

### Optical measurements

A custom microspectroscopy set-up with Thorlabs beam splitters was used to measure microscale reflectivity in the electrochemical cell. Illumination from a 100 W tungsten lamp and light collection was conducted through a 5× air objective (NA 0.14), passing through the top flow cell with a glass cover into the WO_3_ metapixels. A portion of the reflected light was collected by an optical fibre and analysed using a B&WTek CypherX spectrometer. Most photographs were captured using a commercial microscope (Olympus MX50). An iPhone 15 and a Samsung A55 were used to capture the photograph in Fig. 4c (left) and Supplementary Video 1, respectively.

### Arbitrarily selected combinations of RGB pixels

Extended Data Fig. 2 presents an example in which the intermediate regions of RGB pixels contain only cyan and yellow, but not magenta. Specifically, a yellow pixel (*D* = 300 nm, *W* = 200 nm) is observed, between the red (*D* = 320 nm, *W* = 220 nm) and green (*D* = 260 nm, *W* = 200 nm) pixels. Similarly, a cyan pixel (*D* = 280 nm, *W* = 140 nm) appears between the green and blue (*D* = 280 nm, *W* = 120 nm) pixels; however, the magenta pixel (*D* = 260 nm, *W* = 100 nm) is absent from the intermediate region between the red and blue pixels.

By adjusting subpixel spacing, a yellow pixel emerges when the red–green subpixel distance is 240 nm, whereas a cyan pixel forms at a blue–green subpixel distance of 120 nm; however, regardless of how the red–blue subpixel distance is tuned, magenta cannot be generated, contrasting with the results shown in Fig. 2c.

### Refractive index of WO_3_ in on–off states

The refractive index of WO_3_ is obtained from a previously published study^25^. Extended Data Fig. 3 presents both the real (*n*) and imaginary (*k*) components of the refractive index in both the on and off states. In the on state, the real part (*n*) decreases from 2.4 to 2.1, enabling strong Mie scattering in the air. The imaginary part (*k*), which represents optical absorption, remains close to zero, explaining why WO_3_ nanodisks exhibit vivid colours in the air; however, in a liquid environment such as acetonitrile (*n* ≈ 1.33), the refractive index is closer to that of WO_3_, reducing Mie scattering and making the nanodisks more transparent, thus diminishing colour vibrancy, as discussed in the main text. Electrochemical tuning in solution allows WO_3_ nanodisks to switch to the off state, in which the *k-*value significantly increases to 0.05–0.4 in the visible range, leading to strong optical absorption and changing the colour pixels into dark states.

### Optical contrast of red pixel

To compare with the normalized optical contrast shown in Fig. 3c, Extended Data Fig. 4 presents the absolute reflectance variation of red light at a wavelength of 620 nm in response to the applied 40 ms pulse voltage signal (Fig. 3d, left). The red subpixel achieves an optical contrast of more than 48% within 40 ms, and its maximum optical contrast under long-time constant voltage is 50%. This result further confirms WO_3_ metapixels supporting video display.

### Colour memory effect

Owing to the bistability of WO_3_, the retina E-paper exhibits a colour memory effect. In the absence of external energy input, WO_3_ gradually relaxes from its redox states back to the natural state, rather than reverting instantly from a coloured state to a dark state as seen in emissive displays. As shown in Extended Data Fig. 5, once a pixel is switched to the coloured mode, its normalized reflectance remains above 90% for around 150 s under open-circuit conditions. Similarly, when a pixel is switched to the dark mode, it retains a normalized reflectance less than 10% for about 9 s without further energy input. This behaviour enables ultra-low power consumption, as static or minimally changing pixels do not require continuous power. Instead, an approximately 2 ms electronic pulse input (for around 10% optical contrast modulation) is sufficient to maintain the display state.

### CMY pixels in the air

As discussed in the main text, to achieve RGB colours through subtractive colour mixing using CMY primaries, it is crucial to ensure that the intermediate regions between CMY pixels contain RGB pixels. As shown in Extended Data Fig. 6, a blue pixel (*D* = 260 nm, *W* = 120 nm) exists between the cyan (*D* = 260 nm, *W* = 160 nm) and magenta (*D* = 240 nm, *W* = 100 nm) pixels. Similarly, a green pixel (*D* = 260 nm, *W* = 180 nm) is present between the magenta and yellow (*D* = 180 nm, *W* = 180 nm) pixels, while a red pixel (*D* = 200 nm, *W* = 180 nm) appears between the yellow and cyan pixels. By tuning the spacing between subpixels, a blue pixel emerges when the CM subpixel distance is 100 nm, green at an MY subpixel distance of 60 nm, and red at a CY subpixel distance of 60 nm.

### Transfer colour image to nanodisk pixels

Extended Data Fig. 7 illustrates the process of converting the intensity (scaled from 0 to 255) of each CMY pixel into the geometry structure of nanodisk pixels. The intensity of each CMY channel is mapped to the height of its corresponding CMY rectangle as a fraction of the maximum value, representing the percentage of light each CMY subpixel should reflect. The designated nanodisk arrays, which generate CMY colours, are then used to fill these rectangles, approximating the subpixel colour composition. However, due to the small size of the CMY rectangles, the nanodisks cannot achieve perfect spatial coverage, leading to inaccuracies in colour reproduction. This limitation contributes to the imperfections observed in Fig. 4b–d.

### CMY pixels in the electrolyte

The underlying principle remains consistent with CMY pixels in the air. As illustrated in Extended Data Fig. 8, the intermediate regions of CMY colours contain corresponding RGB components. The subpixel distances are *T*_1_ = 40 nm for blue, *T*_2_ = 300 nm for red and *T*_3_ = 60 nm for green. Owing to weaker Mie scattering in the electrolyte, the extinction of red colour becomes weaker, causing a less intense blue colour.

### CMY colour modulation and comparison of colour performance with other devices

Extended Data Fig. 9a (left) illustrates the nanodisk diameters and periodicities for the CMY metapixels alongside their corresponding reflection spectra, which show similar reflectance as RGB pixels. The corresponding geometries are C (*D* = 280 nm, *W* = 20 nm), M (*D* = 220 nm, *W* = 80 nm) and Y (*D* = 300 nm, *W* = 80 nm), and the merged RGB subpixel spacing was adjusted accordingly: B (*T*_1_ = 40 nm), R (*T*_2_ = 300 nm) and G (*T*_3_ = 60 nm).

Extended Data Fig. 9b presents a comparison of the colour gamut coverage among commercial emissive displays, the retina E-paper (under both air and electrolyte conditions), and a commercial colour electrophoretic display. Although the colour gamut of the retina E-paper does not yet match that of emissive technologies, it significantly outperforms existing commercial colour e-paper, highlighting its potential for high-fidelity reflective colour displays.

### Front-illumination integration of retina E-paper into immersive augmented reality–virtual reality optics

Two potential system-level configurations are illustrated in Extended Data Fig. 10, demonstrating how reflective pixels can be effectively illuminated in practical augmented reality–virtual reality devices.

Extended Data Fig. 10a illustrates an optical configuration compatible with conventional virtual reality headsets. In this design, incident light enters from the side and is redirected by a beam splitter through an eyepiece onto the retina E-paper. The reflected light follows the reverse path, passing back through the eyepiece and beam splitter before reaching the eye. This optical arrangement resembles that of a reflective microscopy system and aligns with well-established principles in optical engineering.

Extended Data Fig. 10b depicts an advanced optical architecture compatible with waveguide-based augmented reality–virtual reality systems. In this configuration, light is side-coupled into the device and directed onto the retina E-paper through a beam splitter. The reflected light is then collimated and coupled into the waveguide through input gratings and ultimately delivered to the eye through output gratings. This design enables integration into compact form factors, significantly shortens the eye-to-display distance, and supports a wider field of view—key requirements for achieving immersive and lightweight augmented reality–virtual reality experiences.

### Video‐rate switching red pixel

Supplementary Video 1 visually demonstrates the rapid modulation speed of WO_3_ metapixels by showing the reflectivity change of a red pixel under pulse signals with durations of 1 s, 500 ms, 250 ms and 40 ms. The distinct bright and dark states are clearly visible with evident dwell times, highlighting the full contrast range and switching stability of the device. Although the video was recorded by a cell phone at 50 fps, the camera of the microscope system was used to record the light spot at only 18 fps. As a result, despite the reaction speed exceeding the required for video play (24 fps), intensity variations remain observable.

## Online content

Any methods, additional references, Nature Portfolio reporting summaries, source data, extended data, supplementary information, acknowledgements, peer review information; details of author contributions and competing interests; and statements of data and code availability are available at 10.1038/s41586-025-09642-3.

## Supplementary information

Supplementary Video 1 Video‐rate switching red pixel. This video shows the reflectivity change of a red pixel under pulse signals with durations of 1 s, 500 ms, 250 ms and 40 ms. The distinct bright and dark states are clearly visible with evident dwell times, highlighting the full contrast range and switching stability of the device. Although the video was recorded on a smartphone phone at 50 fps, the camera of the microscope system was used to record the light spot at only 18 fps. As a result, despite the reaction speed exceeding the required for video play (24 fps), intensity variations remain observable.

## Source data

Source Data Figs. 2–4 and Source Data Extended Data Figs. 3–5 and 9.

## Data Availability

The data supporting the findings of this study are available in the paper and its Supplementary Information. Source data are provided with this paper.

## References

[1] Joo, W. J. et al. Creating the ultimate virtual reality display. *Science* **377**, 1377–1378 (2022). 10.1126/science.abq7011 36137048

[2] Lian, Y. et al. Downscaling micro- and nano-perovskite LEDs. *Nature* **640**, 62–68 (2025). 40108467 10.1038/s41586-025-08685-w

[3] Joo, W. J. et al. Metasurface-driven OLED displays beyond 10,000 pixels per inch. *Science* **370**, 459–463 (2020). 33093108 10.1126/science.abc8530

[4] Shin, J. et al. Vertical full-colour micro-LEDs via 2D materials-based layer transfer. *Nature* **614**, 81–87 (2023). 36725999 10.1038/s41586-022-05612-1

[5] Kossyrev, P. A. et al. Electric field tuning of plasmonic response of nanodot array in liquid crystal matrix. *Nano Lett.* **5**, 1978–1981 (2005). 16218721 10.1021/nl0513535

[6] Zang, H. M. et al. Electrophoretic display comprising black, white, red, and yellow particles. *J. Soc. Inf. Disp.* **30**, 387–394 (2022).

[7] Kumar, K. et al. Printing colour at the optical diffraction limit. *Nat. Nanotechnol.* **7**, 557–561 (2012). 22886173 10.1038/nnano.2012.128

[8] Kuznetsov, A. I. et al. Optically resonant dielectric nanostructures. *Science* **354**, aag2472 (2016). 27856851 10.1126/science.aag2472

[9] Proust, J. et al. All-dielectric colored metasurfaces with silicon Mie resonators. *ACS Nano* **10**, 7761–7767 (2016). 27458790 10.1021/acsnano.6b03207

[10] Bao, Y. et al. Full-colour nanoprint-hologram synchronous metasurface with arbitrary hue-saturation-brightness control. *Light Sci. Appl.* **8**, 95 (2019). 31666949 10.1038/s41377-019-0206-2 PMC6813292

[11] Yang, W. et al. All-dielectric metasurface for high-performance structural color. *Nat. Commun.* **11**, 1864 (2020). 32313078 10.1038/s41467-020-15773-0 PMC7171068

[12] Zheng, G. et al. Metasurface holograms reaching 80% efficiency. *Nat. Nanotechnol.* **10**, 308–312 (2015). 25705870 10.1038/nnano.2015.2

[13] Cencillo-Abad, P. et al. Ultralight plasmonic structural color paint. *Sci. Adv.* **9**, adf7207 (2023). 10.1126/sciadv.adf7207 PMC9995036 36888718

[14] Vynck, K. et al. The visual appearances of disordered optical metasurfaces. *Nat. Mater.* **21**, 1035–1041 (2022). 35590040 10.1038/s41563-022-01255-9

[15] Song, M. et al. Versatile full-colour nanopainting enabled by a pixelated plasmonic metasurface. *Nat. Nanotechnol.* **18**, 71–78 (2023). 36471110 10.1038/s41565-022-01256-4

[16] Kim, Y. et al. Active modulation of reflective structural colors. *Chem. Commun.* **58**, 12014–12034 (2022). 10.1039/d2cc04153g 36205156

[17] Peng, J. et al. Scalable electrochromic nanopixels using plasmonics. *Sci. Adv.* **5**, eaaw2205 (2019). 31093530 10.1126/sciadv.aaw2205 PMC6510554

[18] Duan, X. et al. Dynamic plasmonic colour display. *Nat. Commun.* **8**, 14606 (2017). 28232722 10.1038/ncomms14606 PMC5333121

[19] Xiong, K. et al. Plasmonic metasurfaces with conjugated polymers for flexible electronic paper in color. *Adv. Mater.* **28**, 9956–9960 (2016). 27670834 10.1002/adma.201603358

[20] Xiong, K. et al. Video-rate switching of high-reflectivity hybrid cavities spanning all primary colors. *Adv. Mater.* **35**, 2302028 (2023). 10.1002/adma.202302028 37277121

[21] Gugole, M. et al. Comparison of electrodeposited and sputtered tungsten trioxide films for inorganic electrochromic nanostructures. *ACS Appl. Opt. Mater.* **1**, 558–568 (2023).

[22] Moon, C. et al. Active electrochemical high-contrast gratings as on/off switchable and color tunable pixels. *Nat. Commun.* **13**, 3391 (2022). 35697694 10.1038/s41467-022-31083-z PMC9192692

[23] Xiong, K. et al. Switchable plasmonic metasurfaces with high chromaticity containing only abundant metals. *Nano Lett.* **17**, 7033–7039 (2017). 29028347 10.1021/acs.nanolett.7b03665

[24] Xiong, K. et al. Active control of plasmonic colors: emerging display technologies. *Rep. Prog. Phys.* **82**, 024501 (2019). 30640724 10.1088/1361-6633/aaf844

[25] Guo, J. et al. Fast-switching WO_3_-based electrochromic devices: design, fabrication, and applications. *Acc. Mater. Res.* **4**, 438–447 (2023).

[26] Fernández, M. R. et al. Review of display technologies focusing on power consumption. *Sustainability* **7**, 108543–110875 (2015).

[27] Johansson, M. B. et al. Electronic and optical properties of nanocrystalline WO_3_ thin films studied by optical spectroscopy and density functional calculations. *J. Phys. Condens. Matter* **25**, 205502 (2013). 23614973 10.1088/0953-8984/25/20/205502

[28] Xiong, K. et al. Video speed switching of plasmonic structural colors with high contrast and superior lifetime. *Adv. Mater.* **33**, 2103217 (2021). 34448507 10.1002/adma.202103217 PMC11468514

[29] Greeuw, G. et al. Theoretical solution of the transient current equation for mobile ions in a dielectric film under the influence of a constant electric field. *J. Appl. Phys.* **9**, 3371–3375 (1984).

[30] Wen-Cheun Au, B. et al. Effect of film thickness on electrochromic performance of sol-gel deposited tungsten oxide (WO_3_). *Opt. Mater.* **94**, 387–392 (2019).

[31] Bessinger, D. et al. Fast-switching vis-IR electrochromic covalent organic frameworks. *J. Am. Chem. Soc.* **143**, 7351–7357 (2021). 33724793 10.1021/jacs.0c12392 PMC8154512

[32] Zhang, T. et al. Optical-cavity-incorporated colorful all-solid-state electrochromic devices for dual anti-counterfeiting. *Adv. Mater.* **36**, 2402670 (2024). 10.1002/adma.202402670 38663415

[33] Reddy, M. R. et al. Change detection in video using pixel based parametric analysis. *Indian J. Sci. Technol.* **8**, 35 (2015).

[34] Gugole, M. et al. Electrochromic inorganic nanostructures with high chromaticity and superior brightness. *Nano Lett.* **21**, 4343–4350 (2021). 33969987 10.1021/acs.nanolett.1c00904 PMC8289301

[35] Qiu, T. et al. Vision-driven metasurfaces for perception enhancement. *Nat. Commun.* **15**, 1631 (2024). 38388545 10.1038/s41467-024-45296-x PMC10883922

[36] Yin, K. et al. Virtual reality and augmented reality displays: advances and future perspectives. *J. Phys. Photon.* **3**, 022010 (2021).

