# Author Correction: Video‐rate tunable colour electronic paper with human resolution

**DOI:** 10.1038/s41586-025-09975-z

**Published:** 2025-12-02

**Authors:** Ade Satria Saloka Santosa, Yu-Wei Chang, Andreas B. Dahlin, Lars Österlund, Giovanni Volpe, Kunli Xiong

**Affiliations:** 1 Department of Materials Science and Engineering, The Angstrom Laboratory, Uppsala University, Uppsala, Sweden; 2 Department of Physics, University of Gothenburg, Gothenburg, Sweden; 3 Department of Chemistry and Chemical Engineering, Chalmers University of Technology, Gothenburg, Sweden; 4 Present Address: Department of Chemistry, Umeå University, Umeå, Sweden

**Keywords:** Metamaterials, Metamaterials

Correction to: *Nature* 10.1038/s41586-025-09642-3 Published online 22 October 2025

In the version of the article initially published, in Fig. 2a, the *y* axis of the middle panel was shifted upwards and has now been moved down so that “0” is level with the *x* axis. In Fig. 3a, the “RGB pixels” dashed red line has been shortened for clarity. In Extended Data Fig. 2, the *y*-axis label was “W (100 to 200 nm)” but should have been “W (100 to 220 nm)”. In Extended Data Fig. 9a, the *y*-axis labels were shifted downwards and have now been corrected. In Extended Data Fig. 9b, the labels “ln Air” and “ln E” were switched and have now been amended so that the green line represents “ln E” and the blue line “ln Air”. These corrections have been made to the HTML and PDF versions of the article. The original, uncorrected figures are available for comparison in the accompanying Supplementary information.

**Supplementary information** is available in the online version of this amendment.

## Supplementary information

This file contains the original, uncorrected figures.

