# Supplementary material for: Author Correction: Video‐rate tunable colour electronic paper with human resolution
Source: Nature. 2025 Dec 2;648(8094):E16. doi: 10.1038/s41586-025-09975-z (PMC12711559; doi:10.1038/s41586-025-09975-z)
Supplement: Supplementary file 1 — This file contains the original, uncorrected figures. [file 41586_2025_9975_MOESM1_ESM.pdf]

nature portfolio

https://doi.org/10.1038/s41586-025-09975-z

Supplementary information

Author Correction: Video-rate tunable colour electronic paper with human resolution

In the format provided by the authors and unedited

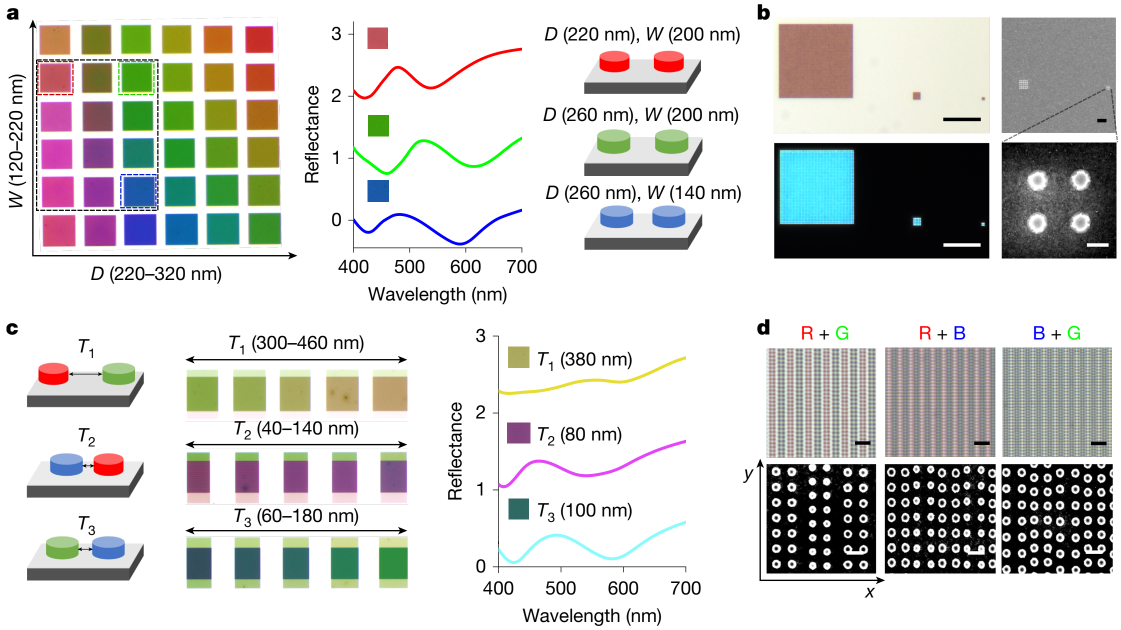

Fig. 2 | Design and characterization of WO_3 metapixels.

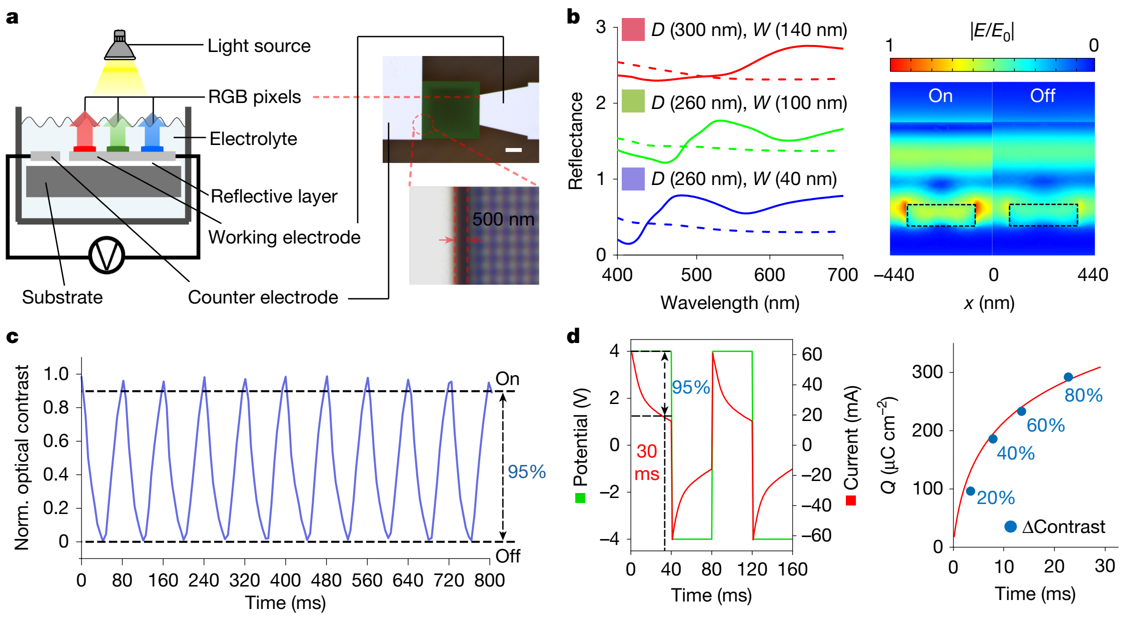

Fig. 3 | Electrochemical modulation of WO_3 metapixels.

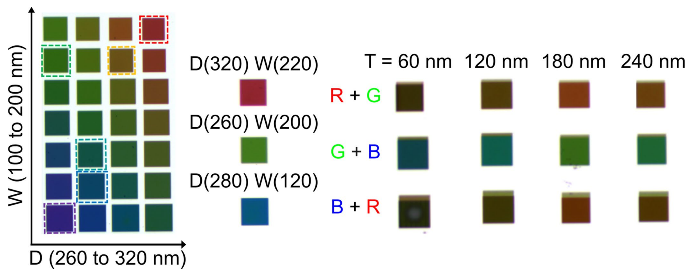

Extended Data Fig. 2 | Arbitrarily selected combinations of RGB pixels.

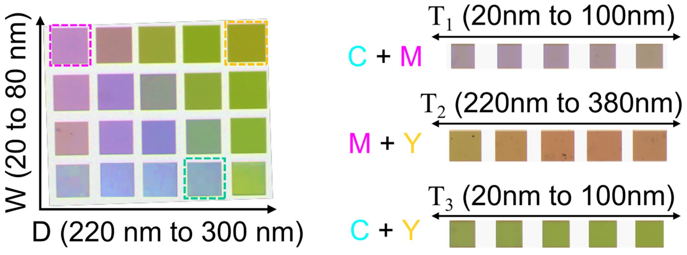

Extended Data Fig. 8 | CMY pixels in the electrolyte.

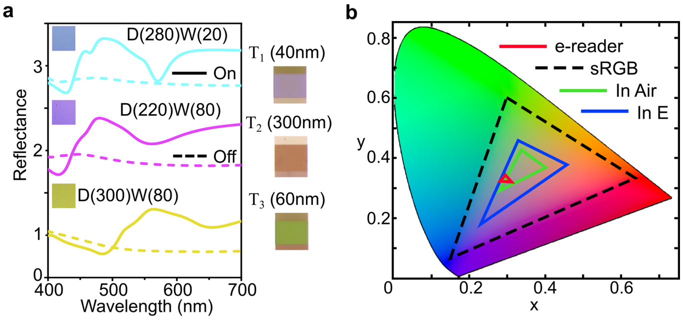

Extended Data Fig. 9 | CMY colour modulation and comparison of colour performance with other devices.
